# Proteomic Analysis of Prostate Cancer FFPE Samples Reveals Markers of Disease Progression and Aggressiveness

**DOI:** 10.3390/cancers14153765

**Published:** 2022-08-02

**Authors:** Vasiliki Lygirou, Konstantinos Fasoulakis, Rafael Stroggilos, Manousos Makridakis, Agnieszka Latosinska, Maria Frantzi, Ioannis Katafigiotis, Christos Alamanis, Konstantinos G. Stravodimos, Constantinos A. Constantinides, Antonia Vlahou, Jerome Zoidakis

**Affiliations:** 1 Biotechnology Division, Biomedical Research Foundation of the Academy of Athens, 4 Soranou Ephessiou Street, 11527 Athens, Greece; vlygirou@bioacademy.gr (V.L.); rstrog@bioacademy.gr (R.S.); mmakrid@bioacademy.gr (M.M.); 2 Department of Urology, Ippokrateio General Hospital of Athens, 114 Vasilissis Sofias Avenue, 11527 Athens, Greece; fasoulakis@gmail.com; 3 Laikon Hospital, 1st Urology Department, Medical School, National and Kapodistrian University of Athens, 17 Agiou Thoma Street, 11527 Athens, Greece; katafigiotis.giannis@gmail.com (I.K.); alamanis@otenet.gr (C.A.); kstravd@med.uoa.gr (K.G.S.); ckonstan@med.uoa.gr (C.A.C.); 4 Mosaiques Diagnostics GmbH, 30659 Hannover, Germany; latosinska@mosaiques-diagnostics.com (A.L.); frantzi@mosaiques.de (M.F.); 5 Department of Biochemistry and Molecular Biology, Faculty of Biology, National and Kapodistrian University of Athens, 15701 Athens, Greece

**Keywords:** prostate cancer, proteomics, biochemical recurrence, biomarkers, FFPE samples, geLC-MS/MS

## Abstract

**Simple Summary:**

Prostate cancer (PCa) is the second most frequently diagnosed type of cancer in men. The lack of tools for accurate risk assessment is causing over-treatment of men with indolent PCa but also delayed detection of metastatic disease and thus high mortality. The aim of our study was to identify proteins related to the progression and aggressiveness of PCa that could serve as potential biomarkers for better risk stratification. To this end, we performed proteomic analysis of Formalin Fixed Paraffin Embedded (FFPE) prostate tissue specimens (*n* = 86) and compared them based on grade groups and biochemical recurrence status. Based on the valuable data generated by these comparisons, we have selected seven proteins (NMP1, *UQCRH*, *HSPA9*, *MRPL3*, *VCAN*, *SERBP1*, *HSPE1*) as common denominators of PCa aggressiveness and persistence that could potentially be used for the development of risk assessment tools. Notably, our observations are largely validated by transcriptomics data and literature.

**Abstract:**

Prostate cancer (PCa) is the second most common cancer in men. Diagnosis and risk assessment are widely based on serum Prostate Specific Antigen (PSA) and biopsy, which might not represent the exact degree of PCa risk. Towards the discovery of biomarkers for better patient stratification, we performed proteomic analysis of Formalin Fixed Paraffin Embedded (FFPE) prostate tissue specimens using liquid chromatography coupled with tandem mass spectrometry (LC-MS/MS). Comparative analysis of 86 PCa samples among grade groups 1–5 identified 301 significantly altered proteins. Additional analysis based on biochemical recurrence (BCR; BCR+ *n* = 14, BCR- *n* = 51) revealed 197 significantly altered proteins that indicate disease persistence. Filtering the overlapping proteins of these analyses, seven proteins (*NPM1*, *UQCRH*, *HSPA9*, *MRPL3*, *VCAN*, *SERBP1*, *HSPE1*) had increased expression in advanced grades and in BCR+/BCR- and may play a critical role in PCa aggressiveness. Notably, all seven proteins were significantly associated with progression in Prostate Cancer Transcriptome Atles (PCTA) and *NPM1NPM1*, *UQCRH*, and *VCAN* were further validated in The Cancer Genome Atlas (TCGA), where they were upregulated in BCR+/BCR-. *UQCRH* levels were also associated with poorer 5-year survival. Our study provides valuable insights into the key regulators of PCa progression and aggressiveness. The seven selected proteins could be used for the development of risk assessment tools.

## 1. Introduction

Prostate cancer (PCa) is the second most frequently diagnosed type of cancer and the fifth leading cause of cancer death in men worldwide [1]. Although PCa is common, the majority of the tumors exhibit indolent or extremely slow progressing disease. On the other hand, approximately 20% of those with localized PCa will develop regional and/or distant metastatic and potentially lethal disease [2].

For about 45% of the PCa patients who present with indolent disease, no immediate treatment is required, and the patients could rather be introduced to an active surveillance (AS) scheme; however, the lack of highly accurate stratification means to timely detect and predict progression, in order to terminate AS and initiate active treatment, substantially blocks the efficient implementation of AS and has, as a result, contributed to significant over-treatment [2]. This is a matter of great concern, as treatment options for PCa are associated with side effects that can have a profound negative impact on quality of life [3].

As for those of intermediate or high-risk PCa, effective risk stratification is very crucial as the prognosis is generally not favorable. In addition, PCa is often a multifocal disease, meaning that numerous tumors can arise within the same prostate. Moreover, in an individual patient, both interfocal and intrafocal genomic and phenotypic heterogeneity are common features. The focal origin of PCa is also thought to influence its metastatic potential [4]. This heterogeneity also predicates an additional challenge associated with identifying molecular biomarkers that might be used for patient risk stratification and direct patient outcomes.

Considering that the aggressiveness of the tumor is defined mainly by the grading system, risk stratification depends on accurate prostate biopsy, even though systematic prostate biopsy misses 21% to 28% of PCa cases and undergrades 14% to 17% [5]. Conventional grading of prostatic adenocarcinoma using the Gleason system is the strongest prognostic factor for clinical behavior and treatment response. The Gleason Score (GS) is calculated by the addition of the two most common grade patterns in prostatectomy samples and the most common grade and highest grade in biopsy samples. This is unique in cancer grading as most other malignancies use the single worst grade pattern observed [6]. Based on the much-revised original GS, a new grading system, consisting of five strata (grade groups 1–5), is the current international standard for pathologists since 2016 and has been used in conjunction with GS [7]. The new grading system is more accurate and simplified compared to the previous ones and has a minimum grade group of 1 (instead of 6 in the GS) with the potential of reduced over-treatment. Importantly, it differentiates GS7 to GS3+4 as grade group 2 and GS4+3 as grade group 3, which have quite a different prognosis [7,8]; however, this method of diagnosis does not recognize the multifocal nature of PCa, especially in the needle biopsy sample, and is highly subjective [9]. Moreover, there is often significant discordance between the grading given to biopsies acquired pre and post-Radical Prostatectomy (RP), with many patients receiving a higher GS following RP [10].

Since its introduction in the late 1980s, Prostate Specific Antigen (PSA) has been the most widely applied test for initial PCa screening, follow-up, and monitoring of recurrence after RP [11]; however, PSA is organ but not cancer-specific; thus, it may be elevated in benign prostatic hypertrophy, prostatitis, and other non-malignant conditions [12]. Conversely, many men may suffer from PCa despite having low serum PSA. In PSA between 3.1 ng/mL and 4.0 ng/mL the risk of PCa is 26.9% [13]. New imaging technology has also been adopted to enhance diagnostic performance (e.g., MRI). Moreover, in recent years, new biomarker assays based on the tissue analysis of multi-gene biomarker panels (such as Decipher, Prolaris, OncotypeDX, and Promark) have been used to classify tumor aggressiveness [14,15,16,17]. These molecular biomarkers may help identify indolent disease graded as GS3+4 or aggressive tumors diagnosed on biopsy as GS3+3. Unfortunately, none of the above technologies fulfill the criteria of a perfect marker regarding sensitivity, specificity, cost-effectiveness, and ease of use in early diagnosis and identification of PCa aggressiveness crucial for efficient treatment [18]. Additionally, the available tests have not been evaluated in prospective randomized trials, and their optimal indication for clinical use remains uncertain; thus, they have not yet been approved by the US Food and Drug Administration (FDA) [2].

Proteomics can be a promising tool for biomarker discovery. A growing number of studies have utilized this approach in PCa research (reviewed in [19,20,21,22]). Proteomic studies in tissue, cell lines, blood, urine, seminal plasma, and exosomes have identified many potential biomarkers for PCa diagnosis, metastasis, aggressiveness, and resistance to treatment. These studies have utilized both untargeted gel-based and gel-free approaches, including two-dimensional gel electrophoresis coupled to mass spectrometry (2-DE-MS) [23,24], liquid chromatography coupled with tandem mass spectrometry (LC-MS/MS), [25,26,27] and capillary electrophoresis coupled to mass spectrometry (CE-MS) [28,29], with LC-MS/MS being the most commonly used approach, as well as targeted mass spectrometry-based approaches [30]. The reproducibility of these findings remains challenging due to the heterogeneity of cancer itself and the various techniques used, but the data generated through these attempts is a great contribution to personalized approaches after more targeted investigations [19]. Among the most frequently emerging proteomic findings are Vinculin (VCL) [31,32,33,34], which is shown to be predictive of PCa metastasis, and Caveolin-1 (CAV1) [35,36,37], which is shown to indicate PCa progression and resistance to docetaxel. Notably, two glycoproteins, N-acylethanolamine acid amidase (NAAA) and protein tyrosine kinase 7 (PTK7) [38] and proneuropeptide-Y (pro-NPY) [39], were shown to be predictive of PCa aggressiveness by tissue proteomic studies and were validated independently by immunohistochemistry. In addition, Promark, which is an 8-marker assay (certified by Clinical Laboratory Improvement Amendments—CLIA) that predicts PCa aggressiveness, is based on a quantitative multiplex proteomics imaging (QMPI) approach [17]. Apart from proteomics, transcriptomics approaches have had a significant contribution to the discovery of PCa biomarkers, as well [40,41,42].

The purpose of this study is the identification of proteins that play a major role in the pathology and progression of PCa and can potentially be used as biomarkers for accurate risk stratification of the disease. Archival Formalin Fixed Paraffin Embedded (FFPE) prostate tissue specimens from patients who had undergone RP at the Ippokrateio Hospital of Athens, Greece, were used in the study. Extracted proteins from the cancerous samples were analyzed by GeLC-MS/MS, followed by statistical and bioinformatics analysis. Comparisons of the proteomic profiles on the basis of tumor grade and biochemical recurrence were conducted to reveal common key players in PCa progression and aggressiveness.

## 2. Materials and Methods

### 2.1. Tissue Samples

Archival FFPE prostate tissue specimens from 86 patients who had undergone RP at the Ippokrateio Hospital, Athens, were used in the study, in line with ethics requirements (the study was approved by the Ethics Committee of the Medical School, National and Kapodistrian University of Athens with protocol number 45/2018). The clinicopathological information associated with these specimens is provided in Table 1. FFPE sections (4 μm thick) from each block were stained with hematoxylin/eosin and examined by the pathologist to localize the site of the tumor and evaluate the Gleason grading. Only tumor regions were selected by microdissection from the adjacent unstained sections. The samples used in the current study correspond to grade group 1 (*n* = 22), grade group 2 (*n* = 27), grade group 3 (*n* = 23), and grade groups 4–5 (*n* = 14). For 65 of the samples, there was also information on biochemical recurrence (BCR, BCR+ *n* = 14 and BCR- *n* = 51).

### 2.2. Sample Preparation for Proteomics

The optimized protocol for protein extraction from FFPE tissue samples was followed exactly as described in [27]. Specifically, from each patient, 3 sections 15 μm thick were used for proteomic analysis in the current study. After deparaffinization and rehydration, the tissue samples were homogenized in FASP lysis buffer (4% SDS, 100 mM DTE, 100 mM Tris-HCl pH 7.6) using a bullet blender homogenizer. In addition, the homogenates underwent 3 cycles of tip sonication for 10 s and 1 h of heating at 90 °C on a heating block to better facilitate the homogenization. Following centrifugation for 10 min at 4000 rcf at room temperature, the supernatants (∼170 μL) were transferred to new 1.5 mL Eppendorf tubes and protease inhibitors were added to a final concentration of 3.6% *v/v* and stored at −80 °C until use.

Prior to protein digestion, the protein extracts were concentrated 10 times (using Amicon Ultra Centrifugal Filters, 3 kDa MW cutoff) with buffer exchange in 50 mM ammonium bicarbonate, pH 8.5. The total amount of the concentrated sample (18–20 μL) was processed following the GeLC-MS protocol exactly as described in [43].

### 2.3. LC-MS/MS Analysis and MS Data Processing

LC-MS/MS analysis was performed on a Dionex Ultimate 3000 UHPLC nanoflow system coupled to a Thermo Q Exactive mass spectrometer. Prior to the analysis, each sample was reconstituted in 12 μL mobile phase A (0.1% formic acid, pH 3.5) and 6 μL loaded into the UHPLC nanoflow system configured with a Dionex 0.1 × 20 mm, 5 μm, 100 Å C18 nano trap column with a flow rate of 5 µL/min. The analytical column was an Acclaim PepMap C18 nano column 75 μm × 50 cm, 2 μm 100 Å with a flow rate of 300 nL/min. The trap and analytical column were maintained at 35 °C. Mobile phase B was 0.1% Formic acid in acetonitrile. The column was washed and re-equilibrated prior to each sample injection. The eluent was ionized using a Proxeon nano spray ESI source operating in positive ion mode. For mass spectrometry analysis, a Q Exactive Orbitrap (Thermo Finnigan, Bremen, Germany) was operated in MS/MS mode. The peptides were eluted under a 240 min gradient from 2% (B) to 80% (B). Gaseous phase transition of the separated peptides was achieved with positive ion electrospray ionization applying a voltage of 2.5 kV. For every MS survey scan, the top 10 most abundant multiply charged precursor ions between m/z ratio 300 and 2200 and intensity threshold 500 counts were selected with FT mass resolution of 70,000 and subjected to HCD fragmentation. Tandem mass spectra were acquired with an FT resolution of 35,000. The normalized collision energy was set to 33 and already targeted precursors were dynamically excluded for further isolation and activation for 30 s with 5 ppm mass tolerance. Raw files were processed with Thermo Proteome Discoverer 1.4 software, utilizing the Sequest search engine and the UniProt human fasta database containing only canonical sequences (downloaded on 16/12/2018, including 20,243 reviewed entries). The search was performed using carbamidomethylation of cysteine as static and oxidation of methionine as dynamic modifications. Two missed cleavage sites, a precursor mass tolerance of 10 ppm, and fragment mass tolerance of 0.05 Da were allowed. The following filters were also used: peptide: Medium Confidence (FDR q value based = 0.05), peptide rank: Maximum rank = 1, peptide grouping: enabled, protein grouping: enabled.

The peak area of precursor ions was used to assess the relative abundance of the identified proteins (label-free method). Protein abundance in each sample was calculated as the sum of all peptide peak areas from the extracted chromatogram, normalized by part per million (ppm): (Protein peak area/total peak area per sample) ×10^6^. For both comparisons (grade groups 1–5 and BCR+/BCR-), only proteins detected in at least 40% of samples in at least one group were considered for statistical analysis. The nonparametric Kruskal–Wallis and Mann−Whitney tests were utilized to define statistical significance in the comparisons grade groups 1–5 and BCR+/BCR-, respectively. Statistical tests and visualizations were conducted in the R language (version 4.0.3). Volcano plots were created with ggplot2 functionality.

### 2.4. Enrichment Analysis

Enrichment analysis was performed in Metascape [44] utilizing the Custom analysis for *Homo sapiens*. For the Enrichment analysis, only the databases Reactome Gene Sets, KEGG Pathway, WikiPathways, Canonical Pathways, and PANTHER Pathway were used to retrieve pathways. For the remaining options, default settings were used. To avoid redundancy, only the summaries of the groups are presented, and the results are simplified based on biological relevance.

### 2.5. Comparison with Prostate Cancer Transcriptome Atlas and the Cancer Genome Atlas

Prostate Cancer Transcriptome Atlas (PCTA) [45] was accessed on 15 July 2022 and the Expression View tool was used for each of the selected proteins separately and for the list of the seven selected proteins against the PCTA dataset by Disease Course. Disease progression was investigated across the following subsets: benign, GS < 7, GS = 7, GS > 7. Metastatic Castration-Resistant PCa (mCRPC) and one-way ANOVA test between subsets were used to define significance.

The Cancer Genome Atlas (TCGA) prostate adenocarcinoma RNA-seq expression and clinical data were downloaded from cBioportal.org (Firehorse Legacy, *n* = 501) on 20/04/2022. Statistical tests and visualizations were conducted in the R language (version 4.0.3). Testing for significance was conducted on the RSEM normalized expression matrix [46], and significance was defined at a Mann–Whitney *p*-value < 0.05. Boxplots were created with ggplot2 functionality.

Survival analysis was performed with the libraries’ survival [47] and survminer [48]. Sample groups of high versus low expression were defined based on a gene-specific median cutoff and were compared for statistical differences in their 5-year survival probability, with the Log–Rank method. A survival plot was created with the function ggsurvplot.

## 3. Results

### 3.1. Discovery of the Most Prominent Changes within the Grade Groups

To reveal the differences in protein abundances among the grade groups of PCa and to highlight potential drivers of malignant progression, we performed high-resolution LC-MS/MS analysis of 86 prostate FFPE tissue samples of grade groups 1–5. Due to the limited number of patients with advanced disease, grade groups 4 and 5 were combined into one group, grade group 4–5, for the analysis.

Proteomic analysis of the tissue samples, following an adjusted preparation protocol for FFPE specimens [27], resulted in the identification of a total of 1262 proteins. The full list of identified proteins is presented in Table S1, and volcano plots for the pair-wise comparisons among the grade groups are presented in Figure S1. Out of these proteins, 301 showed statistically significant differences (Kruskal–Wallis *p*-value < 0.05) among the grade groups 1, 2, 3, and 4–5. Looking into the significant proteomic changes among the grade groups might reveal some of the crucial molecular mechanisms through which the prostate cells become cancerous and progressively aggressive. Based on the enrichment analysis, these proteins are involved mainly in mitochondrial, amino acid, and carbon metabolism but also in processes related to angiogenesis, apoptosis, Rho GTPases signaling, and reactive oxygen species detoxification. The enrichment analysis results and the significantly different proteins that are participating in the pathways are presented in Table S2.

### 3.2. Identification of Proteins Associated with Biochemical Recurrence

**Figure 1:**
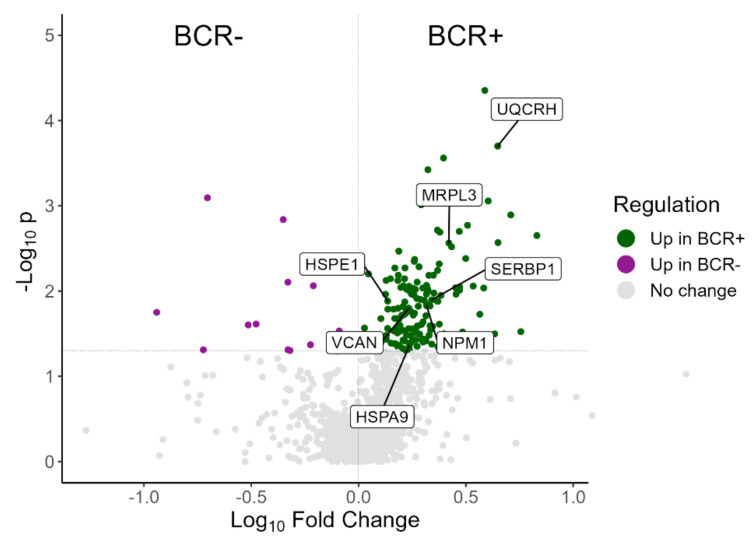
Volcano plot showing the distribution of proteins in the proteomics comparison between BCR+ and BCR-. Green dots represent proteins significantly overexpressed in BCR+; purple dots represent proteins significantly overexpressed in BCR- while grey dots represent proteins with no significant change between BCR+ and BCR-. The seven selected proteins (NMP1, *UQCRH*, *HSPA9*, *MRPL3*, *VCAN*, *SERBP1*, *HSPE1*) are highlighted.

Additional statistical analysis on the MS data of 65 of the prostate tissue samples was also performed to compare the proteomic profiles of patients with biochemical recurrence (BCR+, *n* = 14) with the ones who were recurrence-free (BCR-, *n* = 51). Through this analysis, a total of 1264 proteins were identified, with 197 of them showing a statistically significant difference (Mann–Whitney *p*-value < 0.05) between BCR+ and BCR-. The detailed protein identifications are presented in Table S3. A volcano plot showing results from the differential abundance analysis between BCR+ and BCR- is presented in Figure 1.

Enrichment analysis showed that metabolic processes are highly represented in biochemical recurrence, with pathways related to RNA and protein processing also being a prominent feature. The enrichment analysis results and the significantly different proteins between BCR+ and BCR- that are participating in the pathways are presented in Table S4.

### 3.3. Shortlisting of Proteins Associated with Cancer Aggressiveness and Biochemical Recurrence

In order to highlight proteins that may play a critical role in the aggressiveness and persistence of PCa, we focused on the features found differentially abundant both among grade groups (Kruskal–Wallis *p*-value < 0.05) and between BCR+ and BCR- patients (Mann–Whitney *p*-value < 0.05). Having as a guide the significant changes in BCR+/BCR- comparison, we checked their relative change in abundance within the four grade groups (1, 2, 3, and 4–5). Our focus was on proteins that were either upregulated in BCR+/BCR- and increasing in abundance in ascending order of grade groups or downregulated in BCR+/BCR- and decreasing in abundance in ascending order of grade groups. Apart from the significant change in abundance in BCR+/BCR- comparison and the same expression trend in the two comparisons, another criterion was the significant change in abundance along the grade groups (either Kruskal–Wallis *p*-value < 0.05 and/or Mann–Whitney *p*-value < 0.05 in at least three pair-wise comparisons between the grade groups). While it is understood that protein expression along disease progression and aggressiveness is not necessarily affected in the same way as in cancer relapse, the aforementioned criterion was applied to detect the most probable common denominators of these two processes, within this study.

This filtering method resulted in the selection of seven proteins that would be of interest for further investigation (highlighted in Figure 1). The seven selected proteins and their regulation along the grade groups and BCR status are presented in Table 2. Nucleophosmin (*NPM1NPM1*), Ubiquinol-Cytochrome C Reductase Hinge Protein (or Cytochrome b-c1 complex subunit 6, *UQCRH*), Heat Shock Protein Family A Member 9 (or Stress-70 protein, *HSPA9*), and Mitochondrial Ribosomal Protein L3 (*MRPL3*) are all significantly (Mann–Whitney *p*-value < 0.05) upregulated in BCR+/BCR-, significantly different among the grade groups (Kruskal–Wallis *p*-value < 0.05) and their abundance is increasing in ascending order of grade groups. Additionally, for these four proteins, the pair-wise comparisons between grade groups show some significant differences (Mann–Whitney *p*-value < 0.05), consistent with the comparisons between grade group 4–5 versus grade group 1 and grade group 4–5 versus grade group 2. This observation indicates a direct association of protein levels rise with disease progression. Versican (*VCAN*) and Plasminogen activator inhibitor 1 RNA-binding protein (*SERBP1*) are also significantly upregulated in BCR+/BCR-, significantly different among the grade groups and their abundance is increasing in grade group 3 and grade group 4–5, while it is almost the same for grade group 1 and grade group 2 (*VCAN* grade group 2/grade group 1 = 0.96, *SERBP1* grade group 2/grade group 1 = 0.99). Again, for both *VCAN* and *SERBP1*, the comparisons of grade group 4–5 versus grade group 1 and grade group 4–5 versus grade group 2 supported a significant increase in the protein levels in the advanced versus low PCa grades. The last selected protein, Heat Shock Protein Family E Member 1 (or 10 kDa heat shock protein, *HSPE1*), is also significantly upregulated in BCR+/BCR, its expression rises with ascending order of grade groups, and it shows a significant difference in the pair-wise comparisons grade group 4–5 versus grade group 1, grade group 4–5 versus grade group 2 and grade group 4–5 versus grade group 3, but not in the multi-group comparison (Kruskal–Wallis *p*-value = 0.07).

### 3.4. Cross-Examination of the Selected Proteins in PCTA and TCGA

To further increase the validity of our results, we checked the association between the expression of the seven selected proteins (NMP1, *UQCRH*, *HSPA9*, *MRPL3*, *VCAN*, *SERBP1*, *HSPE1*) and disease progression in PCTA. Interestingly, a significant association with disease progression at the mRNA level was found for all the selected proteins individually, as well as for the whole seven-protein panel (one-way ANOVA *p*-value < 0.05; Table S5). This finding validates the suggested implication of the selected proteins in PCa progression.

Additionally, to further expand the validation of our data, we cross-examined the expression of the seven selected proteins in TCGA. Specifically, TCGA prostate adenocarcinoma mRNA data were downloaded and differential expression analysis between BCR+ (*n* = 58) and BCR- (*n* = 371) patients was conducted for the seven selected proteins. Of those, *NPM1NPM1*, *UQCRH*, and *VCAN* were validated to be significantly upregulated in the BCR+ group compared to the BCR- (Table S6 and Figure S2), as was also described by our proteomics analysis. The rest of the selected proteins were also either upregulated in BCR+ compared to BCR- or practically unchanged within the two groups with no statistical significance.

**Figure 2:**
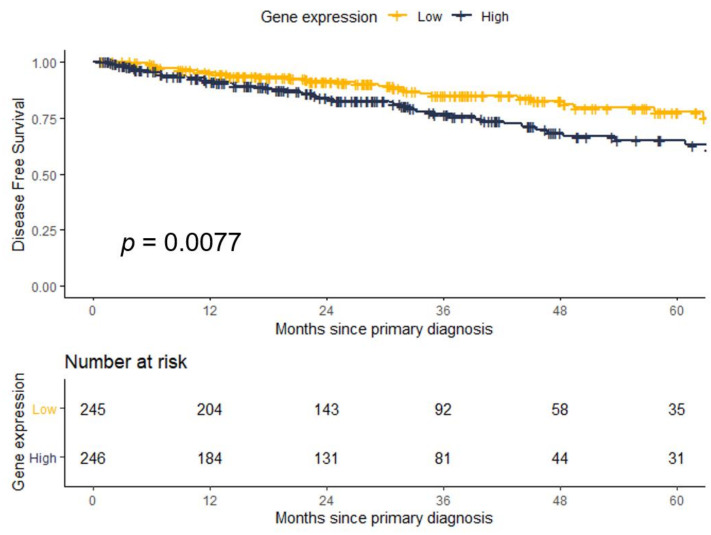
Survival plot of the TCGA prostate adenocarcinoma mRNA data, showing the difference in the decrease in the survival probability between patients with high and low *UQCRH* expression levels. High versus low expression was defined based on a gene-specific median cutoff.

Furthermore, increased expression levels of *UQCRH* were also associated with poorer 5-year survival, as shown in Figure 2.

## 4. Discussion

Although great advances in the research on PCa biology, diagnostics, prognostics, and therapeutics have been made during the last decade, accurate risk assessment and consequential informed management are still missing from the clinical setting. As most of the cases are low-risk PCa, a lack of precise risk assessment tools leads to severe over-treatment [2]. On the other hand, of those who will undergo RP, a great percentage (approximately 20–40%) will develop biochemical recurrence within 10 years [49]. A molecular signature to accurately predict disease aggressiveness at diagnosis and indicate the appropriate mode of action (i.e., active surveillance, chemotherapy, RP, etc.) is thus clearly needed.

In this study, we used a set of well-characterized FFPE samples from PCa patients, spanning from grade group 1 to grade group 5, and performed high-resolution proteomic analysis using an optimized sample preparation protocol. Specifically, we have employed RP excised tissue specimens where the actual cancer spread (pathological stage and grade) can be assessed. This analysis enriches the knowledge on PCa progression by providing a list of proteins and their relative abundance across the disease grades. TCA cycle and overall metabolic reprogramming is a prominent feature represented by the observed protein changes along the progression of PCa in our analysis (shown in Table S2), which is also reflected in the literature [49,50,51,52]. Apart from the metabolic reprogramming, processes related to angiogenesis (neutrophil and platelet degranulation, VEGFA-VEGFR2 signaling pathway) [50,51], apoptosis [52], Rho GTPases [53], and reactive oxygen species signaling [54] are shown to be altered among the grade groups and have been previously linked to PCa and disease progression, which further supports the validity of our findings.

Utilizing the available information, an additional comparative analysis was performed between patients with biochemical recurrence and recurrence-free patients. Enrichment analysis of the significant changes between the two groups suggested differences in the TCA cycle [55,56], mitochondrial metabolism [57,58], angiogenesis [50,51], Rho GTPases [53], extracellular matrix organization [59], and ALK signaling [60], which have been associated with PCa aggressiveness and/or recurrence before. Significant differences based on the observed protein changes were also predicted in the BARD1 pathway, Interleukin-12 signaling, and a group of processes related to RNA and protein processing (Table S4, that, to the best of our knowledge, have not yet been associated with PCa aggressiveness and may merit further investigation.

Focusing on the significant findings in these comparisons, seven proteins (NMP1, *UQCRH*, *HSPA9*, *MRPL3*, *VCAN*, *SERBP1*, *HSPE1*) were selected based on their consistency in all comparisons performed; hence, that could potentially be indicative of PCa progression and aggressiveness. Further enhancing the validity of these results, all of the selected proteins were significantly associated with disease progression in PCTA at the mRNA level, both separately and as a seven-feature panel. Moreover, for three of these proteins, *NPM1NPM1*, *VCAN*, and *UQCRH*, we were able to validate their relative increase in BCR+ compared to BCR- using prostate adenocarcinoma mRNA data in TCGA. In addition, *NPM1NPM1*, *VCAN*, *HSPA9*, *SERBP1*, and *HSPE1* have been studied previously in the context of PCa (summarized below) and have been correlated with disease aggressiveness (but not biochemical recurrence), although the rest (*UQCRH* and *MRPL3*) have not yet been associated with PCa (to the best of our knowledge) and constitute novel findings.

*NPM1NPM1*, which is a nucleocytoplasmic shuttling protein, was found overexpressed in PCa compared to healthy adjacent tissue and it was shown to favor migration, invasion, and colony forming. Moreover, it was shown that NMP1 was positively regulated by the ERK1/2 (Extracellular signal-Regulated Kinases 1/2) kinase phosphorylation in response to EGF (Epidermal Growth Factor) stimulus, which is critical for PCa progression [61]. Furthermore, Destouches et al. demonstrated that although total and phosphorylated *NPM1NPM1* were found overexpressed in castration-resistant PCa, the multivalent pseudopeptide N6L was able to inhibit tumor growth both in vitro and in vivo when used either alone or in combination with the standard-of-care treatments for advanced PCa [62].

*VCAN* is a large extracellular matrix proteoglycan that accumulates in tumor stroma and plays a key role in malignant transformation and tumor progression, through regulation of cell adhesion, proliferation, apoptosis, migration, angiogenesis, invasion, and metastasis. Increased *VCAN* expression has been observed in a wide range of malignant tumors and has been associated with both cancer relapse and poor patient outcomes in breast, prostate, and many other cancer types [63]. Arichi et al. identified *VCAN* as a marker of clinical outcomes in docetaxel-resistant castration-resistant PCa patients treated with docetaxel and thalidomide. In addition, the effect of docetaxel and thalidomide therapy on cell viability was the same as the effect of docetaxel plus *VCAN* siRNA, suggesting that targeting *VCAN* could be a potential therapeutic strategy in docetaxel-resistant PCa [64].

*HSPA9* is upregulated in many tumors compared to healthy tissue and is an inhibitor of complement-dependent cytotoxicity, protecting cells from antibody-based immune therapy. In a recent retrospective study of 636 RP patients, *HSPA9* expression was associated with an increased risk of high-grade adenocarcinoma and biochemical failure after salvage therapy, as detected by microarrays [65]. *HSPA9* is also part of a 12-biomarker panel that predicted PCa aggressiveness (surgical Gleason and TNM stage) and lethal outcome robustly in both high- and low-Gleason areas, despite biopsy-sampling error [66].

*SERBP1* is an RNA binding protein involved in mRNA maturation and translation regulation. It also regulates one-carbon metabolism and epigenetic modification of histones, and increased *SERBP1* expression in cancers such as leukemia, ovarian, prostate, liver, and glioblastoma is correlated with poor patient outcomes [67]. In addition, *SERBP1* was found upregulated in PCa tissues and was significantly associated with tissue metastasis and Gleason score [68].

*HSPE1* is a major heat shock protein that functions as a chaperonin. Aberrant expression of this protein has been associated with the progression of PCa [69] but also with other types of cancer, including colorectal adenocarcinoma [70], hepatocellular carcinoma [71], and non-small cell lung cancer [72].

*UQCRH* is a multisubunit transmembrane complex that is part of the mitochondrial electron transport chain, which drives oxidative phosphorylation. It was found overexpressed in BCR+ patients compared to BCR- in both our dataset and in TCGA analysis. Moreover, based on the TCGA data, we showed that the expression levels of *UQCRH* were associated with 5-year survival, with lower levels predicting a better survival probability. Even though no data on PCa are available, *UQCRH* was found overexpressed in hepatocellular carcinoma. This overexpression correlated with larger tumor size, poorer differentiation or vascular invasion, and shorter overall and disease-free survival [73]. Elevated expression of *UQCRH* was also observed in breast cancer tumors compared to normal breast tissue counterparts [74]; however, its role in cancer progression is somewhat controversial since in renal cell carcinoma, the expression of *UQCRH* levels in cancer tissues was lower than normal adjacent tissues [75] and low *UQCRH* levels were associated with high TNM stage, poor survival, and early recurrence [76].

*MRPL3* is a component of the 39S subunit of the mitochondrial ribosome and is part of diagnostic and/or prognostic signatures for breast cancer [77], pancreatic carcinoma [78], and lung adenocarcinoma [79]. In all of these studies, *MRPL3* levels were higher in cancer tissues compared to normal ones.

Taking into consideration the relatively increasing abundance of the seven selected proteins across the grade groups, their increased levels in BCR+ compared to BCR- and the available research on their involvement in (prostate) cancer, it is suggested that the selected proteins are promising candidates for further investigation as potential PCa aggressiveness markers.

Although the data presented here are of great value, we acknowledge that our study has its limitations. Although RPs are more accurately graded compared to biopsies and some prognostic signatures developed with RPs have been useful for active surveillance in prostate needle biopsy settings, our results might be more relevant to patients after primary treatment. In addition, the sample size per group is relatively small (average *n*/grade group = 21 ± 5) especially considering disease heterogeneity. Additionally, the comparison between BCR+ and BCR- is not optimally balanced (14 BCR+, 51 BCR-). This is due to the fact that a low percentage of patients develop biochemical recurrence. To overcome these limitations, we applied a 40% filter on the identifications (present in at least 40% of the samples of a group in at least one group) and focused on the statistically significant differences. In addition, the selected proteins were derived from the overlap of the significant changes from both comparisons and were cross-examined with PCTA, TCGA, and the literature. A more detailed expression analysis of the seven selected proteins in normal prostate and different PCa forms, by immunohistochemistry, is additionally planned to further support the validity of the potential biomarkers.

## 5. Conclusions

In the present study, proteomic analysis of RP FFPE samples from PCa patients of all grade groups provides a valuable source of proteins involved in disease pathology and progression. Taking into consideration the biochemical recurrence status of the samples (where available), we were able to identify seven proteins (NMP1, *UQCRH*, *HSPA9*, *MRPL3*, *VCAN*, *SERBP1*, *HSPE1*) as common denominators of PCa aggressiveness and persistence, which could potentially be used for the development of risk assessment tools. As for most of these proteins, there is previous evidence of an association with PCa progression, metastasis, recurrence, and resistance to therapy. Our observations are largely validated by independent transcriptomics data. Our findings constitute a strong foundation for further validations towards biomarker development and identification of novel therapeutic targets.

## Figures and Tables

**Table 1:** Clinicopathological characteristics of the patients. Values are presented as average ± standard deviation or *n*. Kruskal–Wallis tests were used for continuous variables in grade group comparisons, Mann–Whitney test was used for continuous variables in BCR comparisons, and Fisher exact test was used for categorical variables.

Cohort Characteristics	Grade Group 1	Grade Group 2	Grade Group 3	Grade Group 4–5	*p*-Value	BCR+	BCR-	*p*-Value
total sample size (*n*)	22	27	23	14		14	51	
age (years)	67 ± 6.2	65 ± 5.9	67 ± 5.8	71 ± 4.8	5.58 × 10^−2^	67 ± 7.0	67 ± 6.3	8.89 × 10^−1^
serum PSA (ng/mL)	6.8 ± 2.70	8.0 ± 4.94	11.0 ± 7.62	20.2 ± 36.35	1.81 × 10^−2^	9.7 ± 6.64	7.5 ± 2.83	3.47 × 10^−1^
body mass index (kg/m^2^)	25.9 ± 3.96	26.3 ± 2.66	27.8 ± 2.94	25.8 ± 2.01	2.08 × 10^−1^	26.6 ± 3.66	26.2 ± 3.21	7.72 × 10^−1^
tumor stage								
pT1 (*n*)	2	1	0	0	1.40 × 10^−3^	0	3	9.64 × 10^−2^
pT2 (*n*)	16	14	9	2		4	29	
pT3 (*n*)	4	11	14	12		10	18	
pT4 (*n*)	0	1	0	0		0	1	
lymph nodes								
N0 (*n*)	22	27	22	12	7.59 × 10^−2^	13	51	2.15 × 10^−1^
N1 (*n*)	0	0	1	2		1	0	
metastasis								
M0 (*n*)	21	26	23	14	8.30 × 10^−1^	13	50	3.87 × 10^−1^
M1 *(n*)	1	1	0	0		1	1	
*n* of patients per grade group								
Grade group 1 (*n*)						4	17	4.47 × 10^−1^
Grade group 2 (*n*)						3	19	
Grade group 3 (*n*)						4	10	
Grade group 4–5 (*n*)						3	5	

**Table 2:** List of the seven selected proteins that present with significant protein abundance changes in the proteomics comparison between BCR+ and BCR- and in the advanced versus early PCa grades. Significant *p*-values (*p*-value < 0.05) are marked in blue.

**Heading**	***VCAN***	***NPM1NPM1***	***UQCRH***	***SERBP1***	***HSPA9***	***MRPL3***	***HSPE1***
Ratio BCR+/BCR-	1.68	2.00	4.45	2.19	1.80	2.59	1.37
Mann–Whitney *p*-value in BCR+ vs. BCR-	1.83 × 10−^2^	1.89 × 10^−2^	1.99 × 10^−4^	1.27 × 10^−2^	1.24 × 10^−2^	3.66 × 10^−3^	1.19 × 10^−2^
Kruskal–Wallis *p*-value	5.89 × 10^−4^	1.01 × 10^−2^	1.16 × 10^−2^	1.86 × 10^−2^	3.20 × 10^−2^	3.28 × 10^−2^	6.66 × 10^−2^
Grade group 1 Average	57.39	77.31	5.60	25.42	56.82	11.33	535.94
Grade group 2 Average	55.18	78.39	6.50	25.11	63.96	18.20	565.24
Grade group 3 Average	193.80	79.77	8.98	34.74	99.01	26.33	705.18
Grade group 4–5 Average	273.18	188.05	33.66	63.35	121.72	56.49	872.93
Mann-Whitney *p*-value in Grade group 2 vs. Grade group 1	9.59 × 10^−1^	7.19 × 10^−1^	9.17 × 10^−1^	8.83 × 10^−1^	4.32 × 10^−1^	6.23 × 10^−1^	6.68 × 10^−1^
Mann–Whitney *p*-value in Grade group 3 vs. Grade group 2	1.29 × 10^−2^	9.84 × 10^−1^	7.81 × 10^−1^	6.60 × 10^−1^	3.95 × 10^−1^	7.62 × 10^−1^	7.00 × 10^−1^
Mann–Whitney *p*-value in Grade group 4–5 vs. Grade group 3	2.59 × 10^−1^	5.13 × 10^−3^	2.39 × 10^−2^	3.11 × 10^−2^	1.21 × 10^−1^	3.26 × 10^−2^	3.61 × 10^−2^
Mann–Whitney *p*-value in Grade group 3 vs. Grade group 1	2.50 × 10^−2^	5.87 × 10^−1^	6.85 × 10^−1^	5.99 × 10^−1^	1.17 × 10^−1^	7.67 × 10^−1^	8.84 × 10^−1^
Mann–Whitney *p*-value in Grade group 4–5 vs. Grade group 1	1.01 × 10^−3^	4.03 × 10^−3^	8.07 × 10^−3^	7.66 × 10^−3^	7.83 × 10^−3^	9.73 × 10^−3^	1.72 × 10^−2^
Mann–Whitney *p*-value in Grade group 4–5 vs. Grade group 2	5.48 × 10^−4^	4.32 × 10^−3^	4.90 × 10^−3^	4.15 × 10^−3^	2.14 × 10^−2^	2.29 × 10^−2^	1.74 × 10^−2^

## Data Availability

The raw and search files from the proteomic analysis are available on MassIVE (Mass Spectrometry Interactive Virtual Environment) open access repository (https://massive.ucsd.edu/ProteoSAFe/static/massive.jsp) under the identifier MSV000089707 and on ProteomeXchange open access repository (http://www.proteomexchange.org/) under the identifier PXD034838.

## Supplementary Materials

The following supporting information can be downloaded at: https://www.mdpi.com/article/10.3390/cancers14153765/s1; Table S1: List of the total protein identification in the comparison among the grade groups. Table S2: List of significantly enriched pathways based on the statistically significant differences (Kruskal-Wallis *p*-value < 0.05) in the comparison among the grade groups. Table S3: List of the total protein identification in the comparison between BCR+ and BCR-. Table S4: List of significantly enriched pathways based on the statistically significant differences (Mann-Whitney *p*-value < 0.05) in the comparison between BCR+ and BCR-. Table S5: One-way ANOVA test results from the PCTA on the association of the seven selected proteins with prostate cancer progression (disease course). Table S6: The seven selected proteins in the transcriptomics comparison between patients with biochemical recurrence (*n* = 58) and patients without biochemical recurrence (*n* = 371) in TCGA data. Figure S1: Volcano plots showing the distribution of proteins in the pair-wise comparisons within the grade groups. G1, G2, G3, and G4-G5 refer to grade groups 1, 2, 3, and 4–5, respectively. Mann−Whitney test was utilized to define statistical significance. The seven selected proteins (NMP1, *UQCRH*, *HSPA9*, *MRPL3*, *VCAN*, *SERBP1*, *HSPE1*) are highlighted. Volcano plots on the comparisons between grade groups A. 2 and 1; B. 3 and 1; C. 4–5 and 1; D. 3 and 2; E. 4–5 and 2; F. 4–5 and 3. Figure S2: Boxplots showing the mRNA levels for *NPM1NPM1*, *UQCRH*, and *VCAN* in BCR- and BCR+, as detected in TCGA. These were the only three out of the seven selected features that showed statistically significant change (Mann-Whitney *p*-value < 0.05) in BCR+/BCR- in TCGA, in agreement with the proteomics. BCR- is presented in red and BCR+ is presented in blue.
