# Supplementary material for: Proteomic Analysis of Prostate Cancer FFPE Samples Reveals Markers of Disease Progression and Aggressiveness
Source: Cancers (Basel). 2022 Aug 2;14(15):3765. doi: 10.3390/cancers14153765 (PMC9367334; doi:10.3390/cancers14153765)

Supplementary Figure S1. Volcano plots showing the distribution of proteins in the pair-wise comparisons within the grade groups. G1, G2, G3 and G4-G5 refer to grade groups 1, 2, 3, and 4-5, respectively. Mann−Whitney test was utilized to define statistical significance. The seven selected proteins (NMP1, UQCRH, HSPA9, MRPL3, VCAN, SERBP1, HSPE1) are highlighted. Volcano plots on the comparisons between grade groups **A.** 2 and 1; **B.** 3 and 1; **C.** 4-5 and 1; **D.** 3 and 2; **E.** 4-5 and 2; F. 4-5 and 3.

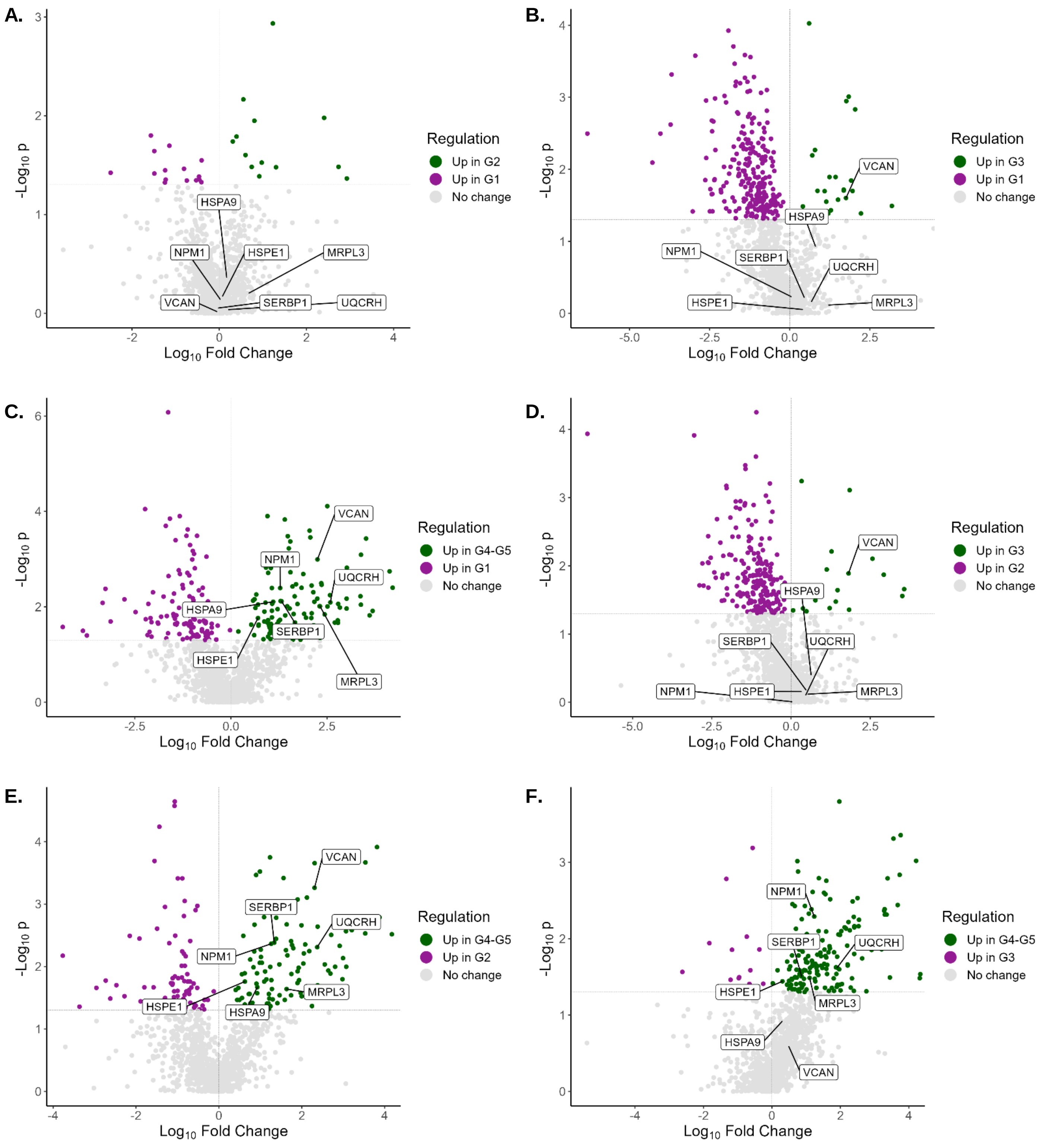

Supplementary Figure S2. Boxplots showing the mRNA levels for NPM1, UQCRH and VCAN in BCR- and BCR+, as detected in TCGA. These were the only three out of the seven selected features that showed statistically significant change (Mann-Whitney p-value < 0.05) in BCR+/BCR- in TCGA, in agreement with the proteomics. BCR- is presented in red and BCR+ is presented in blue.

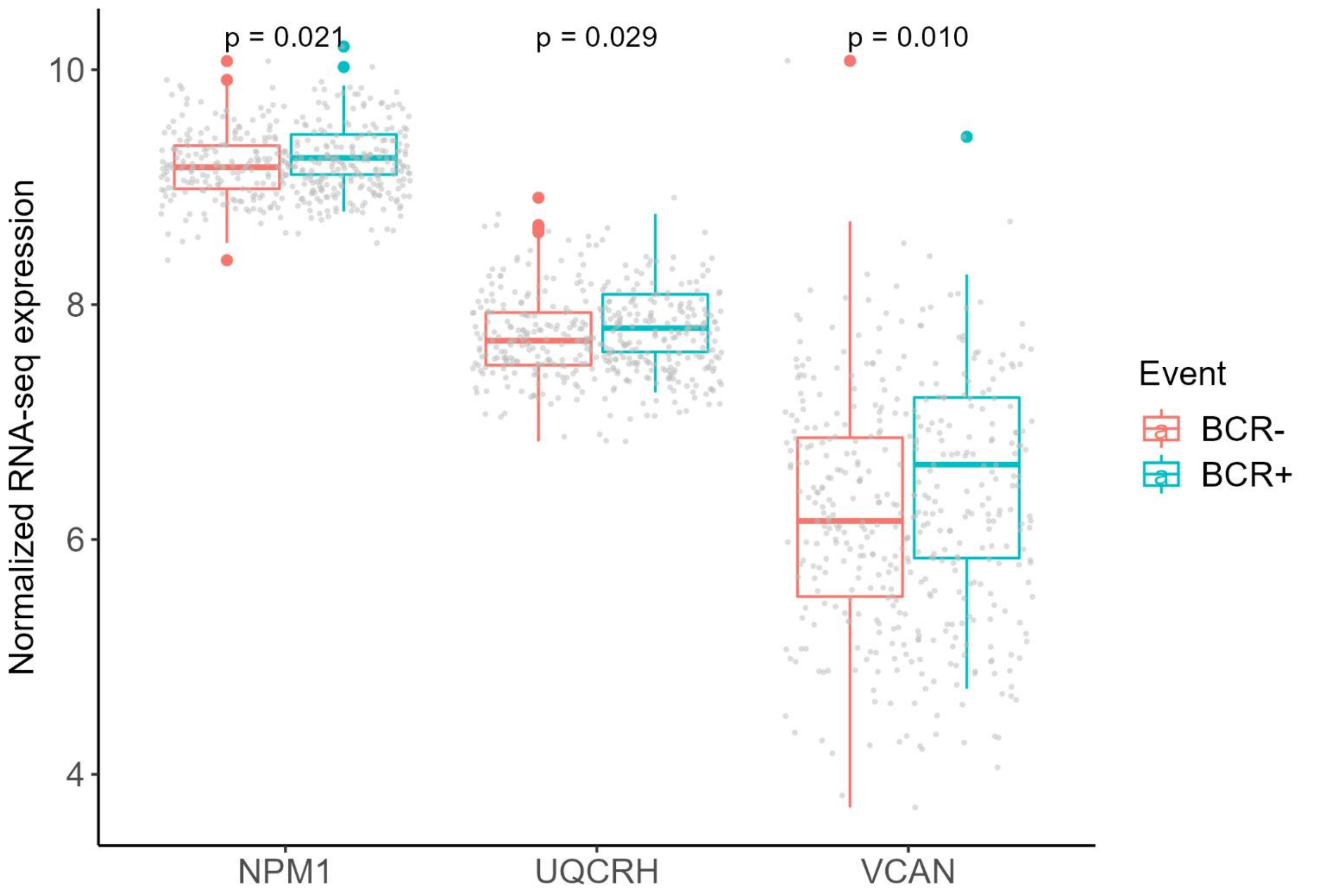

Supplement: Supplementary file 1 [file cancers-14-03765-s001.zip › Supplementary figures.pdf]
